# Organic Acids: The Pools of Fixed Carbon Involved in Redox Regulation and Energy Balance in Higher Plants

**DOI:** 10.3389/fpls.2016.01042

**Published:** 2016-07-15

**Authors:** Abir U. Igamberdiev, Alexander T. Eprintsev

**Affiliations:** ^1^Department of Biology, Memorial University of Newfoundland, St. John’sNL, Canada; ^2^Department of Biochemistry and Cell Physiology, Voronezh State UniversityVoronezh, Russia

**Keywords:** aconitate, citrate, fumarate, malate, organic acids, oxalate, redox level, tricarboxylic acid cycle

## Abstract

Organic acids are synthesized in plants as a result of the incomplete oxidation of photosynthetic products and represent the stored pools of fixed carbon accumulated due to different transient times of conversion of carbon compounds in metabolic pathways. When redox level in the cell increases, e.g., in conditions of active photosynthesis, the tricarboxylic acid (TCA) cycle in mitochondria is transformed to a partial cycle supplying citrate for the synthesis of 2-oxoglutarate and glutamate (citrate valve), while malate is accumulated and participates in the redox balance in different cell compartments (via malate valve). This results in malate and citrate frequently being the most accumulated acids in plants. However, the intensity of reactions linked to the conversion of these compounds can cause preferential accumulation of other organic acids, e.g., fumarate or isocitrate, in higher concentrations than malate and citrate. The secondary reactions, associated with the central metabolic pathways, in particularly with the TCA cycle, result in accumulation of other organic acids that are derived from the intermediates of the cycle. They form the additional pools of fixed carbon and stabilize the TCA cycle. *Trans-*aconitate is formed from citrate or *cis-*aconitate, accumulation of hydroxycitrate can be linked to metabolism of 2-oxoglutarate, while 4-hydroxy-2-oxoglutarate can be formed from pyruvate and glyoxylate. Glyoxylate, a product of either glycolate oxidase or isocitrate lyase, can be converted to oxalate. Malonate is accumulated at high concentrations in legume plants. Organic acids play a role in plants in providing redox equilibrium, supporting ionic gradients on membranes, and acidification of the extracellular medium.

## Introduction

Organic acids are the product of incomplete oxidation of photosynthetic assimilates. They can either be converted back to carbohydrates or undergo terminal oxidation yielding CO_2_ and H_2_O. Their carbon skeletons can also be used for biosynthesis of amino acids. The “intermediate” nature of organic acids determines the flexibility of their role as important players in the maintenance of redox balance, production and consumption of ATP, support of protonic and ionic gradients on membranes, and acidification of extracellular spaces.

Organic acids are formed within metabolic cycles and pathways and represent the transitory or stored forms of fixed carbon. Considering a metabolic system at steady state, the reaction rate of every step can be determined as the concentration of molecules in this step, divided by the mean time that one molecule needs to move the next step. This mean time can be designated as transient time for this step, and according to Easterby (1996) the reaction rate can be written as

(1) $$v_{\text{i}}\;=\;S_{\text{i}}/\tau_{\text{i}}$$

where *v_i_* is the reaction rate in the *i*-th step, *S_i_* is the concentration of the metabolites and *t_i_* is the transient time for this step. The concentrations of the metabolites at steady state, when all reactions proceed with the same rate (*v*_i_ = *J*), can be written from the previous equation for every step as:

(2) $$S_{\text{i}}\;=\;Jt_{\text{i}},\text{i}\;=\;1,\ldots ,n$$

where *J* is the steady-state metabolic flux in the cycle (Fridlyand and Scheibe, 1999). Thus, organic acids accumulate if the transient time for their conversion is long. The increase of *t_i_* for an enzyme leads to the accumulation of metabolite at this step. This can be due to low activity of the enzyme or its low concentration. In the Calvin–Benson cycle, the concentrations of intermediates are established according to the reaction rates of corresponding enzymes and the transient times of metabolite conversions (Fridlyand and Scheibe, 1999). The stability of a cycle’s structure is connected with the existence of transient pools of intermediates that are accumulated during the operation of the cycle. Accumulation of organic acids in steady state conditions is always determined by the transient times of their conversion.

**FIGURE 1:**
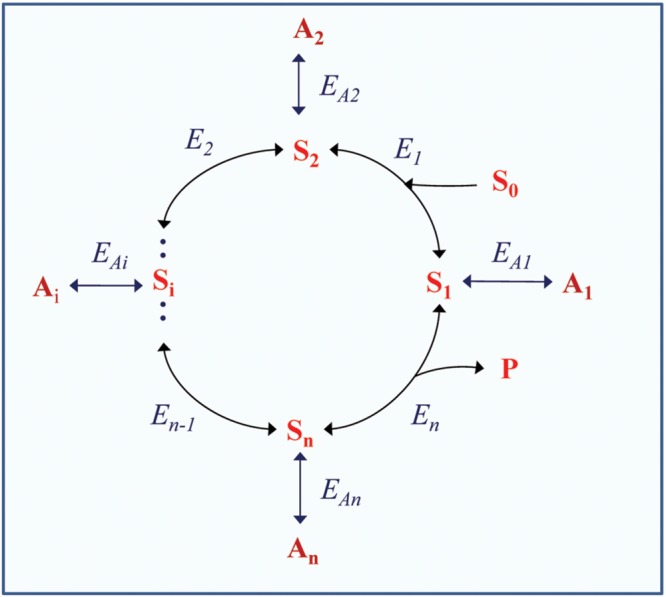
**Scheme of a cyclic enzymatic pathway with secondary pools of intermediate derivatives.** S_0_ is the initial substrate; S_1_, S_i_, and S_n_ are cycle intermediates; P is the product; E_1_, E_i_, and E_n_ are the enzymes catalyzing corresponding steps within the cycle; A_1_, A_2_, A_i_, A_n_ are intermediate derivatives; E_A1_, E_A2_, E_Ai_, E_An_ are the enzymes catalyzing corresponding side reactions forming the secondary pools. The initial scheme of the cycle (without side reactions) is adapted with modifications from Fridlyand and Scheibe (1999).

The structure of a metabolic cycle can also involve the secondary (auxiliary) pools of intermediate derivatives that are formed from the intermediates via their transformations that in many cases are reversible (**Figure 1**). These pools can stabilize operation of the cycle, ensure its maintenance when the main substrate is not efficiently supplied, and provide operation of the cycle as “incomplete” in order to modify its function in response to environmental changes and disturbances. The transient times of secondary intermediates are usually long, their conversion is slow (e.g., for oxalate or malonate), which determines high rates of their accumulation in particular conditions in certain species. The mentioned intermediate derivatives can acquire special functions in metabolism and increase its flexibility.

Organic acids represent the category of compounds that contain carboxylic groups negatively charged at neutral pH and to a lesser extent at acidic pH. Their functions therefore can change depending on pH of the solution, and their excretion can result in the release of protons and, therefore, in acidification of soil, apoplast and vacuole. In this review, we discuss the accumulation and functional role of organic acids related mainly to the operation of the tricarboxylic acid (TCA) cycle and photorespiration. We do not analyze in detail the relation of organic acid and amino acid metabolism and their formation in glycolysis, methylglyoxal pathway and lysine catabolism.

## Incomplete Tricarboxylic Acid Cycle and the Operation of Malate and Citrate Valves

The TCA cycle can regulate the redox and energy level in the cell and supply substrates for amino acid synthesis through the operation of malate and citrate valves, the intensities of which depend on the transformation of the TCA cycle into an open structure. Also the secondary derivatives of the TCA cycle intermediates form corresponding pools that can feed the cycle and stabilize its operation. Differences in the organization of metabolic pathways in different plants will lead to accumulation of different organic acids.

**FIGURE 2:**
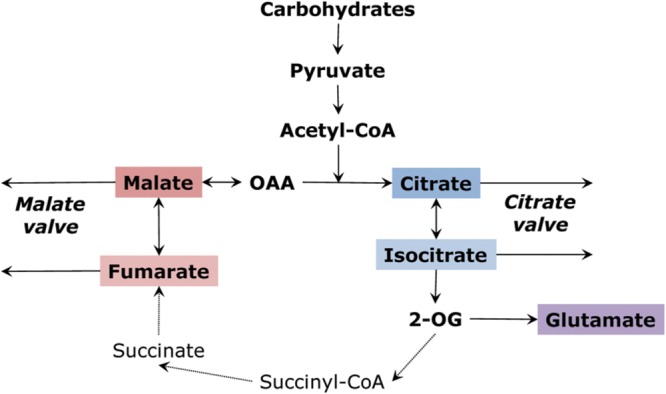
**Operation of the tricarboxylic acid cycle in a “two branches” mode.** Malate and citrate valves are the result of outflow of organic acids from the two branches.

The TCA cycle can operate either in the complete (closed) or the incomplete (open) mode. The concept of the incomplete TCA cycle was initially suggested by Chen and Gadal (1990), Hanning and Heldt (1993) and Gardeström et al. (2002), proven by studying kinetics of NAD- and NADP-dependent isocitrate dehydrogenases by Igamberdiev and Gardeström (2003), confirmed by using stable isotopes of carbon by Tcherkez et al. (2009), and studied via flux modeling (Sweetlove et al., 2010). Transformation to the incomplete cycle is achieved due to the increase of redox level taking place, in particular, in the light, especially in photorespiratory conditions (Igamberdiev et al., 2001a; Igamberdiev and Gardeström, 2003; Gardeström and Igamberdiev, 2016). In the “non-cyclic” partial TCA cycle, one branch produces citrate which can be transformed to isocitrate, 2-oxoglutarate or their derivatives (including glutamate), while the other branch produces malate (or fumarate and even succinate) that can be exported from mitochondria and accumulated in vacuoles (**Figure 2**). The vacuole plays an important role as an intermediary store of malate, citrate and other organic acids; therefore, vacuolar transport functions as a very well-regulated process. This results in the cytosolic pool of malate (and likely of other organic acids) remaining quite stable, with the vacuole serving as a buffer (Meyer et al., 2010a, 2011).

Malate, in many plants, is the most accumulated acid and can participate in the transfer of redox equivalents between cell compartments (Geigenberger and Fernie, 2011; Maurino and Engqvist, 2015). Malate fulfills many functions in plant cells, one of which includes its role as an osmolyte and an anion, compensating the positive charge of potassium, particularly important in stomatal responses (Meyer et al., 2010b). The “malate valve” in photosynthetic cells is driven by NADPH formed by photosynthetic electron transport (Krömer and Scheibe, 1996). Malate accumulation is facilitated due to the equilibrium of malate dehydrogenase [OAA][NADH]/[Malate][NAD^+^] ∼ 2.8 × 10^-5^ (Guynn et al., 1973), established in the conditions of the physiological values of NADH/NAD^+^ ratio, which are equal to 10^-3^ in the cytosol and 10^-2^–10^-1^ in mitochondria in photosynthetic cells (Heineke et al., 1991; Igamberdiev et al., 2001a). In the light, the inhibition of succinate dehydrogenase and fumarase increases malate levels (Eprintsev et al., 2013, 2016; Daloso et al., 2015). The ratio of malate to oxaloacetate (OAA) is an indicator of the reduction level of NAD. The chloroplast NADP-malate dehydrogenase activity is generally lower than NAD-malate dehydrogenase (located in mitochondria, cytosol, peroxisomes, cell wall, some activity in chloroplasts) and does not contribute significantly to the accumulation of malate, except in special cases (photosynthetic induction etc., see Igamberdiev et al., 1998). The conversion of malate to pyruvate via NAD-malic enzyme in mitochondria (Tronconi et al., 2015) and NADP-malic enzyme in the cytosol (Gerrard Wheeler et al., 2009) is under the control of fumarate. When fumarate accumulates, the conversion of malate to pyruvate is facilitated. This takes place during light-enhanced dark respiration (LEDR) (Igamberdiev et al., 2001b).

Malate dehydrogenase, due to its high concentration in mitochondria and other cell compartments and high turnover number, has an important function of regulating the NADH/NAD^+^ ratio and keeping NADH at a low level. This prevents the inhibition of several enzymes resulting from the buildup of NADH and facilitates metabolic respiratory flux. The role of malate dehydrogenase as a “buffering” enzyme relieving NADH inhibition was demonstrated for the operation of glycine decarboxylase (Bykova et al., 2014) and pyruvate dehydrogenase (Igamberdiev et al., 2014a) complexes. This operation of malate dehydrogenase under conditions of saturation by malate represents an important function of the malate valve in the regulation of redox levels in different cell compartments.

Other organic acids associated with malate conversions are fumarate, succinate, and the derivatives of OAA (e.g., malonate). Although succinate dehydrogenase and fumarase genes are downregulated in the light via phytochrome A (Eprintsev et al., 2013, 2016; Igamberdiev et al., 2014b) and the enzyme proteins are inhibited by high redox levels (Daloso et al., 2015), even the decreased activity of fumarase in the light can result in the accumulation of fumarate at very high concentrations (more than 10 mM), e.g., in *Arabidopsis* (Chia et al., 2000). Operation of succinate dehydrogenase in the reverse direction is thermodynamically possible at high redox levels in conjunction with the operation of Complex I supplying electrons from NADH via ubiquinone (Grivennikova et al., 1993), however, this fumarate reductase activity has not been yet demonstrated in plants. Succinate accumulation under hypoxia (Menegus et al., 1989) is also due to higher reduction states in mitochondria and a possible fumarate reductase reaction; however, it may be connected with the inhibition of succinate dehydrogenase and operation of a γ-aminobutyric shunt (António et al., 2016), activated due to a decrease in the cytosolic pH (Crawford et al., 1994).

The “citrate valve” is driven by the increased reduction level in mitochondria (Igamberdiev and Gardeström, 2003), connected, in the light, with photorespiratory metabolism in C_3_ plants. This valve supplies the anabolic reduction power in the form of NADPH, formed by the activity of cytosolic isocitrate dehydrogenase, and the conversion of 2-oxoglutarate to glutamate, which, in some cases, becomes the main product of the partial TCA cycle (Tcherkez et al., 2009). It is likely that citrate is exported from mitochondria preferentially as compared to isocitrate and 2-oxoglutarate. The aconitase equilibrium is strongly displaced toward citrate, and the mitochondrial aconitase, as compared to the cytosolic form, has a lower affinity to citrate and a higher affinity to isocitrate that favors citrate accumulation (Eprintsev et al., 2015a). Citrate export is more than ten times as fast as the export of 2-oxoglutarate in isolated pea leaf mitochondria and twice as fast in spinach leaf mitochondria (Hanning and Heldt, 1993). The citrate carrier from pea is inactive with isocitrate, although the carrier from maize exhibited isocitrate transport capacity (McIntosh and Oliver, 1992). ^13^C nuclear magnetic resonance studies confirmed that in intact leaves in the light citrate is a major mitochondrial product (Gout et al., 1993). Isocitrate and 2-oxoglutarate are then formed from the exported citrate in the cytosol via the cytosolic aconitase and NADP-isocitrate dehydrogenase which activities are high and usually exceed the activities of the mitochondrial isoforms of these enzymes (Eprintsev et al., 2015a).

Cellular concentrations of citrate have a major impact on nuclear-encoded transcript abundance in *Arabidopsis* (Finkemeier et al., 2013). Isocitrate or other citrate analogs does not exhibit similar effects. The effects include regulation of the TCA cycle, nitrogen and sulfur metabolism, DNA synthesis and were found to be similar to observed after biotic stress treatments and the gibberellin biosynthesis inhibitor paclobutrazol. It is suggested that the changes in carboxylic acid abundances can be perceived in *Arabidopsis* by as yet unknown signaling pathways. Previously reported activation of transcription of the alternative cyanide-resistant oxidase in plants by citrate (Vanlerberghe and McIntosh, 1996) can be a part of the more general function of citrate at the transcriptional level. This regulation can be achieved via inhibition of aconitase by reactive oxygen and nitrogen species in stress conditions (Hausladen and Fridovich, 1994; Gupta et al., 2012), or by suppression of isocitrate oxidation at the increased redox level (Igamberdiev and Gardeström, 2003).

Operation of the citrate and malate valves takes place between mitochondria, cytosol and other organelles. This corresponds to the localization of several enzymes participating in the TCA cycle also in the cytosol and in other organelles. The universality of malate as a redox transport compound determines localization of different isoforms of NAD-malate dehydrogenase in mitochondria, cytosol, peroxisomes, plastids and apoplast (Gietl, 1992) as well as the presence of light-activated NADP-malate dehydrogenase in chloroplasts, NAD-malic enzyme in mitochondria and NADP-malic enzyme in plastids and cytosol (Drincovich et al., 2001). Aconitase is present in mitochondria, cytosol and likely absent from peroxisomes (Eprintsev et al., 2015a). While NAD-dependent isocitrate dehydrogenase is located in mitochondria, NADP-dependent isocitrate dehydrogenase is found in mitochondria, cytosol, peroxisomes and plastids (Randall, 1981; Corpas et al., 1999). Fumarase in several plants has not only mitochondrial but also cytosolic localization, and its cytosolic form can be linked to utilization of the glyoxylate cycle products in maize (Eprintsev et al., 2014), in allocation of photosynthates and growth on high nitrogen in *Arabidopsis* (Pracharoenwattana et al., 2010). Although the main route of succinate metabolism is the mitochondrial complex II, its oxidation can be linked to the reduction of nitrate on plasma membrane (Wienkoop et al., 1999) and to the malonate-insensitive activity of succinate oxidase in glyoxysomes of cereals (Igamberdiev et al., 1995). The coordinated operation of isoenzymes of the TCA cycle enzymes in mitochondria, cytosol and other organelles balances the intercompartmental redox regulation, supplies intermediates for biosynthetic pathways and provides the flexibility of the TCA cycle operation in open versus closed form. An important role in the coordination of regulation of two branches of the TCA cycle may belong to the non-glyoxysomal isocitrate lyase, which operates in the cytosol at low pH and activated by manganese (Eprintsev et al., 2015b). It may link the TCA cycle with glyoxylate/glycine metabolism, and, therefore, either supply glyoxylate for glycine and oxalate biosynthesis or utilize the photorespiratory glyoxylate in the reverse (synthase) reaction (Igamberdiev and Lea, 2002).

## Di- and Tricarboxylic Acids in C_4_ and Cam Photosynthesis

C_4_ and CAM photosynthetic metabolism represent a special role for organic acids as intermediate pools of fixed carbon. While the product of CO_2_ fixation is PGA in the Benson–Calvin cycle, the fixation of CO_2_ by phosphoenolpyruvate carboxylase results in the formation of OAA which is then reduced to malate in C_4_ and CAM. Malate, thus, becomes the central metabolite in C_4_ and CAM photosynthesis, playing a double role as a product in the fixation of CO_2_ and a redox equivalent carrier. Its decarboxylation, via NAD- or NADP-malic enzyme, delivers carbon to the Benson-Calvin cycle. In some plants, OAA is preferentially transaminated to aspartate, and CO_2_ delivery occurs via PEP carboxykinase (Burnell and Hatch, 1988).

Although OAA and malate are the primary products of C_4_ and CAM photosynthesis, the plants of these photosynthetic types can intensively accumulate organic acids of the citrate branch. Citrate (or isocitrate) can be the most abundant stored form of carbon in several plants. These plants maintain their metabolic balance by transforming a part of stored malate to citrate via the TCA cycle (Lüttge, 1988). In *Bryophyllum calycinum* (Pucher et al., 1949) during morning hours, citrate and isocitrate were present in equal to the concentration of malate, while in evening hours their concentration exceeded the concentration of malate by fivefold, and isocitrate was the prevalent acid. Citrate and malate accumulated equally in the CAM cycling plant *Euphorbia milii* (Herrera, 2013). Independent fluctuations of malate and citrate, related to photosynthesis and respiration respectively, were found in the CAM tree *Clusia hilariana* (Miszalski et al., 2013).

Citrate/isocitrate synthesis and utilization can be analyzed in a model that considers the open structure of the TCA cycle in the light and closed structure in the dark (Cheung et al., 2014). Accumulation of citrate in the dark via degradation of starch through glycolysis, pyruvate dehydrogenase and citrate synthase, according to this model, provides a carbon-neutral route for transferring ATP and reducing power from the light phase to the dark phase, conserving the net carbon required to support dark metabolism. The role of citrate and isocitrate in C_4_ and CAM metabolism needs further investigation, and the link of the C_4_ cycle with the C_6_ organic acids as the intermediates of the stored fixed carbon is important for understanding the whole structure of metabolism and bioenergetics of C_4_ and CAM plants. Cheung et al. (2014) also consider the link between the two branches of the TCA cycle in the light via isocitrate lyase. The form of this enzyme that operates outside of the glyoxylate cycle (Igamberdiev et al., 1986; Eprintsev et al., 2015b) may be important for balancing of the fluxes of succinate and isocitrate and linking the TCA cycle via glyoxylate to glycine and serine metabolism. This form is activated by lower pH and manganese ions (Eprintsev et al., 2015b). The whole metabolic structure of C_4_ and CAM photosynthesis can be considered a consequence of transformation of malate and citrate valves into the open structure of the TCA cycle to accommodate carbon fixation via PEP carboxylase.

## Trans-Aconitate – A Stored Tricarboxylic Acid Pool

**FIGURE 3:**
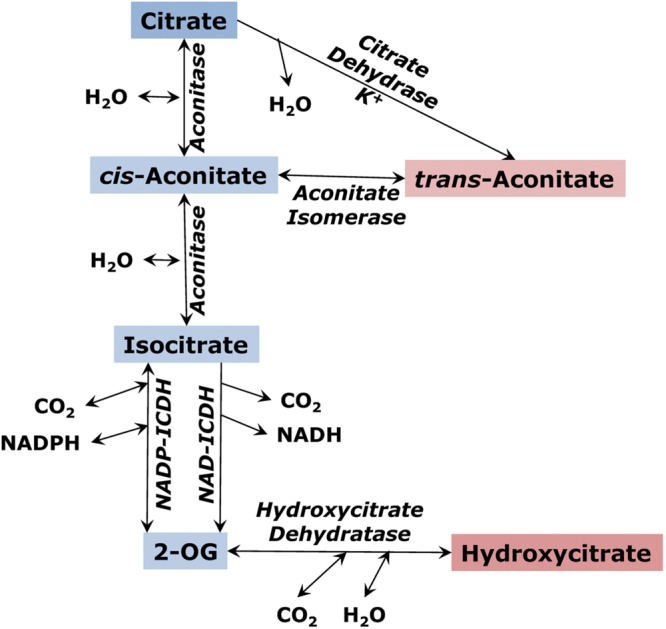
**The citrate branch of the tricarboxylic acid cycle and the formation of *trans*-aconitate and hydroxycitrate as alternative pools of tricarboxylic acids**.

The tricarboxylic branch of the TCA cycle involves citrate, which is formed as a product of citrate synthase reaction, and its isomer D-*threo*-isocitrate, which is further decarboxylated to 2-oxoglutarate by either an NAD- or NADP-dependent isocitrate dehydrogenase. The conversion of citrate to isocitrate by aconitase involves the formation of *cis*-aconitate, a TCA that, being converted to the *trans-*form, can exist as a stable pool. Another TCA derived from the TCA cycle intermediates is hydroxycitrate (**Figure 3**). The aconitase reaction establishes an equilibrium that includes three organic acids: citrate, *cis*-aconitate and isocitrate. The aconitase equilibrium is shifted toward citrate; however, the ratio between its intermediates depends on the concentration of magnesium, affinity of aconitase to its substrates and other factors (Blair, 1969; Eprintsev et al., 2015a). In the TCA cycle *cis*-aconitate appears as a low-concentration intermediate, however, it can be accumulated in plants as the more stable isomer, *trans*-aconitate (Stout et al., 1967). Its concentration is above 1% in cereals (MacLennan and Beevers, 1964). In some plants like *Asarum europaeum* (Krogh, 1971) or aconitum (Otten and Mehltretter, 1970) *trans*-aconitate is a prevalently accumulating organic acid. It is a strong inhibitor of aconitase (Eprintsev et al., 2015a); therefore, it needs to be compartmented from both mitochondrial and cytosolic forms of this enzyme and transferred to vacuole.

**FIGURE 4:**
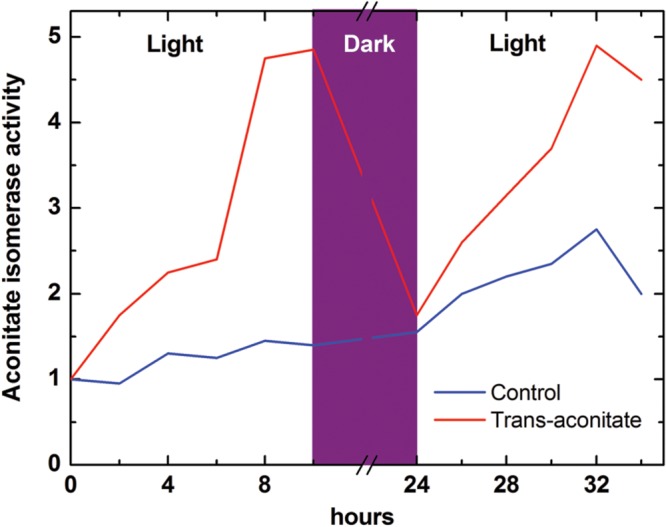
**Induction of aconitate isomerase by *trans*-aconitate in the light in barley leaves (following Zemlyanukhin and Eprintsev, 1979).** Aconitate isomerase activity was monitored as incorporation of the label from 6-^14^C-*trans*-aconitate (synthesized from 6-^14^C-citrate) into CO_2_ via aconitate isomerase, aconitase and isocitrate dehydrogenase reactions; the control value (at time 0 for leaves) is taken as 1. The induction of aconitate isomerase in roots by *trans*-aconitate was light-independent (not shown). Both in leaves and roots aconitate isomerase induction was suppressed by the inhibitor of translation on 80S ribosomes cycloheximide (not shown).

*Trans*-aconitate is produced from *cis*-aconitate by aconitate isomerase (EC 5.3.3.7), an enzyme found in both microorganisms and plants (Thompson et al., 1997). Aconitate isomerase was detected first in bacteria (Rao and Altekar, 1961). MacLennan and Beevers (1964), by using a radioactive label, found that *trans*-aconitic acid is utilized by maize roots and therefore claimed that aconitate isomerase exists in higher plants. The location of aconitate isomerase in maize was more that 90% cytosolic, with the enzyme having a pH optimum of 8.0, a *K*m (*trans*-aconitate) of 3.3 mM, and a molecular weight of 80 kDa (Tkacheva and Zemlyanukhin, 1993). In barley leaves, aconitate isomerase was induced on the cytosolic ribosomes (cycloheximide-sensitive) by externally applied *trans*-aconitate occurring in the light but not in the dark (**Figure 4**), while in the roots its synthesis was induced by *trans*-aconitate in darkness (Zemlyanukhin and Eprintsev, 1979). This can indicate the role of *trans*-aconitate metabolism during photosynthesis in leaves.

Another enzyme participating in *trans*-aconitic acid synthesis is citrate dehydrase (Brauer and Teel, 1981, 1982). This enzyme, in the presence of potassium, catalyzes the dehydration of citrate with the formation of *trans*-aconitate. The reaction is similar to that of aconitase, except with respect to the stereo-configuration of the product and its apparent inability to be converted to isocitrate. In this regard, the reaction produces a dead-end metabolic product, although its possible reversibility can return *trans*-aconitate back to metabolism. The kinetics of two molecular forms of K-dependent citrate dehydrase in leaves of maize (*Zea mays* L.) has been reported (Brauer and Teel, 1982). The isozymes were found to be compartmented in mitochondria (25%) and the cytosol (75%). The mitochondrial form exhibited hyperbolic kinetics with respect to both citrate and potassium with the *K*m values of 2.3 and 12 mM, respectively, and the pH optimum of 7.2. The cytosolic form exhibited the hyperbolic kinetics with respect to citrate (*K*m 0.6 mM) but the sigmoidal kinetics with respect to potassium. This may account for the positive correlation between leaf potassium and *trans*-aconitic acid in certain grasses (Clark, 1968). According to the reaction, citrate dehydrase represents an alternative pathway of citrate conversion in plants resulting in the formation of the compound that can be returned back to metabolism via the action of aconitate isomerase. The formation of *trans*-aconitate by citrate dehydrase in mitochondria takes place upon the increase of citrate concentration, while in the cytosol it is stimulated in a higher extent by the increase in potassium concentration. It is still not certain of what kind of enzyme it could be. There were no studies on identification of this protein or its genes, and no further investigation of its role in plant metabolism.

The action of the enzymes citrate dehydrase and aconitate isomerase results in plant cells in the alternative conversion of citrate, as compared to its primary metabolism toward isocitrate formation (aconitase) or acetyl-CoA and OAA production (ATP-citrate lyase). Similarly to fumarate, aconitic acid absorbs ultraviolet in the region at 240 nm. This light is completely absorbed by the ozone layer; therefore it is unlikely that this can have any physiological significance. *Trans*-aconitate may act as an antifeedant against brown plant hoppers (Katsuhara et al., 1993). It also induces tetany in ruminant animals by depleting free magnesium in blood (Bohman et al., 1969), which is caused at even higher intensity by the product of *trans*-aconitate conversion by bacteria, tricarballylate (Russell and Van Soest, 1984). Efficient binding of free magnesium displaces aconitase equilibrium, making it less directed toward citrate (Blair, 1969); it also affects operation of glycolysis and other processes (Igamberdiev and Kleczkowski, 2011). At zero magnesium, the citrate/isocitrate ratio established by aconitase is equal 9, while at 1 mM Mg^2+^ it is 21, and at 2 mM Mg^2+^ it is 36 (Blair, 1969); this means that in the conditions of effective binding of magnesium by *trans*-aconitate, organic acid metabolism is more directed toward isocitrate formation and, therefore, for amino acid biosynthesis. *Trans*-aconitate itself is an efficient inhibitor of the mitochondrial form of aconitase while its inhibition of the cytosolic form of aconitase is lower (Eprintsev et al., 2015a). Taken together, these data indicate the importance of *trans*-aconitate accumulation for regulation of the cytosolic and mitochondrial aconitases and possibly for directing metabolism toward isocitrate formation in the cytosol. Future studies should result in identification and characterizations of the genes of citrate dehydrase and aconitate isomerase in plants, their origin and distribution among plant species.

## Hydroxycitrate and its Link to 2-Oxoglutarate

Hydroxycitrate was first discovered in sugar beet juice (Lippman, 1883) where it is present at low concentration. Later it was found at higher quantities in the species of Hibiscus (Griebel, 1942) and in fruits of *Garcinia cambogia* (up to 30% of dry weight) (Lewis and Neelakantan, 1965). Hydroxycitrate has two asymmetric centers and can exist as four stereo-isomers, however, only two of them were found in plants (Stallings et al., 1979). The first isomer, (+) *allo*-hydroxycitrate, was found in Hibiscus, while the second, (-) hydroxycitrate, was isolated from fruits of Garcinia. Shchiparev and Soldatenkov (1972) demonstrated that (+) *allo*-hydroxycitrate is readily metabolized not only in Hibiscus but also in maize, Phaseolus and other plants.

The enzyme interconverting hydroxycitrate and 2-oxoglutarate in Micrococcus bacteria has a molecular weight of 112 kDa, a *K*m (hydroxycitrate) of 0.35 mM, and a pH optimum of 7.7–8.3 (Zemlyanukhin and Zemlyanukhin, 1996). Such enzyme was also demonstrated by the same authors in the seedlings of *Hibiscus cannabinus*, but its purification was not achieved due to its instability. It may be similar to the enzyme dihydroxyacid dehydratase (EC 4.2.1.9) involved in metabolism of branched-chain amino acids (Kanamori and Wixom, 1963) and catalyzing dehydration of 2,3-dihydroxy-3-methylvalerate and 2,3-dihydroxyisovalerate to corresponding ketoacids, 2-keto-3-methylvalerate and 2-ketoisovalerate respectively (Oliver et al., 2012). This enzyme may be important for gametophyte development and for resistance to salinity stress in *Arabidopsis* (Zhang et al., 2015). Flint and Emptage (1988) and Pirrung et al. (1989) characterized this enzyme in spinach leaves and showed that it contains a [2Fe-2S] cluster, which resembles some similarity to aconitase. Isocitrate dehydrogenase (mostly NADP-dependent) exhibits some activity with the isomer of hydroxycitrate from Garcinia but not from Hibiscus (Plaut et al., 1975), forming 4-hydroxy-2-oxoglutarate. (-) Hydroxycitrate is a strong inhibitor of ATP-citrate lyase, which may be important for the suppression of lipid biosynthesis (Jena et al., 2002).

## Accumulation of Malonate

Malonate is intensively accumulated in the plants belonging to legume family (Fabaceae). The pioneering work on malonate in plants (Bentley, 1952) demonstrated that it is usually accumulated at the levels of 0.5–2 mg per g of fresh weight in legumes. In *Phaseolus vulgaris* it is the main accumulating acid. Fougère et al. (1991) demonstrated that the highest concentration of malonate in alfalfa (*Medicago sativa*) plants is observed in bacteroids. Malonate is essential for symbiotic nitrogen metabolism (Kim, 2002). It exceeds 3.3% of dry weight in kidney vetch (*Anthyllis vulneraria* subsp. *polyphylla*), which corresponds to >10 mM, it accumulates at the level of 0.5% of dry weight in *Astragalus dasyanthus*, 0.2% in *Trifolium pratense*, i.e., in millimolar concentrations (Nikolaeva and Makeev, 1984). Malonate accumulates also at significant concentrations in the representatives of Umbelliferae (Apiaceae) family (e.g., in Anthriscus and Apium), where its concentration reaches 1 mg per g fresh weight (Bentley, 1952). While malonate is a potent inhibitor of succinate dehydrogenase and its exogenous application is toxic for plants (Chen et al., 2011), the efficient compartmentalization of this acid would prevent its strong inhibitory effect on metabolism.

de Vellis et al. (1963) and Shannon et al. (1963) studied malonate biosynthesis in bush bean roots and purified the enzyme catalyzing oxidative decarboxylation of OAA. The nature of this enzyme, however, was not further investigated. Ivanov and Zemlyanukhin (1991) traced the utilization of radiolabelled malate in different gaseous media and established the increase of malonate production from malate under hypoxia. This confirms that accumulation of malonate in these conditions can take place via the malate branch of the TCA cycle, presumably in the reaction of OAA decarboxylation.

**FIGURE 5:**
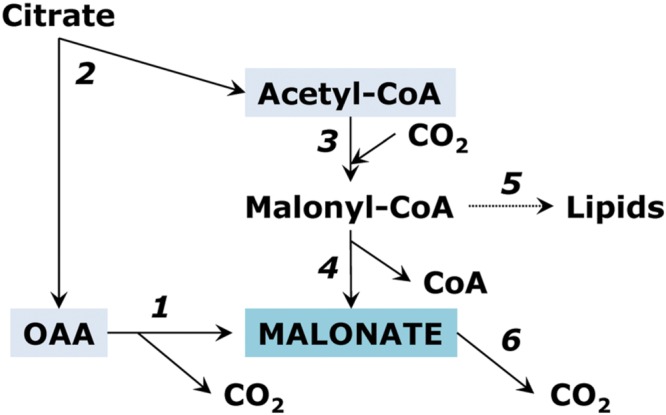
**Reactions of malonate metabolism.** (1) oxaloacetate decarboxylase reaction; (2) ATP-citrate lyase; (3) acetyl-CoA carboxylase; (4) malonyl-CoA hydrolase reaction; (5) biosynthesis of fatty acids; (6) oxidation of malonate to CO_2_.

Stumpf and Burris (1981) studied malonate biosynthesis in roots of soybean seedlings and showed that this compound is not a dead end product of metabolism. The pathway of malonate biosynthesis in young soybean root tissue takes place via acetyl-CoA carboxylase. It may involve further hydrolysis of malonyl-CoA, which is similar to the hydrolysis of acetyl-CoA leading to the formation of acetate in plants (Zemlyanukhina et al., 1986; Zeiher and Randall, 1990). In the pathway initiated by acetyl-CoA carboxylase, malonate serves as a direct precursor of neutral lipids. Groeneveld et al. (1992) demonstrated that malonate is the most effective precursor not only of neutral lipids but also of cardenolides (cardiac glycosides) via glucoevatromonoside in foxglove (*Digitalis lanata*). However, the pathway of lipid biosynthesis should start not necessarily from the carboxylation of acetyl CoA but can also involve directly the malonate pool. Chen et al. (2011) studied malonyl-CoA synthetase in *Arabidopsis* and showed that, although in plants as well as in animals, malonyl-CoA is commonly derived from acetyl-CoA by acetyl-CoA carboxylase (EC 6.4.1.2), it can be produced directly from malonic acid by malonyl-CoA synthetase (EC 6.2.1.14). Malonate can be oxidized with the formation of CO_2_, oxalate, glyoxylate and formate. This reaction is catalyzed by manganese peroxidase in the absence of hydrogen peroxide (Hofrichter et al., 1998). The pathways of malonate metabolism are schematically presented on **Figure 5**.

## Accumulation of Oxalate

Oxalate is one of the most actively accumulating organic acids in many plants such as spinach, beet, Oxalis. It can be present as a soluble form but also forms Ca-oxalate crystals in vacuoles, characterized by different shapes and different Ca^2+^ to oxalate ratios (Franceschi and Nakata, 2005). Oxalate crystal formation has a function of the storage of large amounts of Ca^2+^ in vacuoles, and prevention of the increase of Ca^2+^ level in other compartments, as well as the maintenance of the level of concentration gradient between the vacuole and the cytosol. Cereals, including wheat, are also efficient in oxalate accumulation, and its concentration in seeds may exceed 50 mg g^-1^, while in wheat bran it is 10 times higher (Siener et al., 2006).

The most evident way of oxalate accumulation is the oxidation of glyoxylate (Richardson and Tolbert, 1961; Igamberdiev et al., 1988; Yu et al., 2010). Glyoxylate can be formed during photorespiration in the reaction catalyzed by glycolate oxidase, or it can be produced by isocitrate lyase. Glycolate oxidase reaction was first demonstrated by Kolesnikov (1948) and Tolbert et al. (1949); later a possibility of glycolate dehydrogenase reaction was also suggested for higher plants (Bari et al., 2004). The enzymatic mechanism of glycolate and glyoxylate conversion is similar to that of lactate, the acid produced in high amounts during anaerobic fermentation.

**FIGURE 6:**
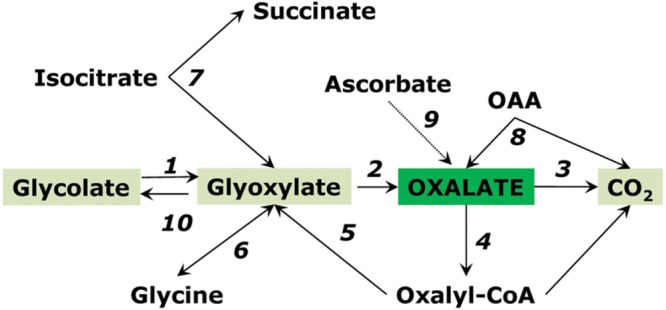
**Pathways of oxalate conversion in plants.** Enzymes: (1) glycolate oxidase; (2) glycolate oxidase (side reaction); (3) oxalate oxidase; (4) oxalyl-CoA synthetase; (5) oxalyl-CoA reductase; (6) aminotransferase; (7) isocitrate lyase; (8) oxaloacetate decarboxylase; (9) ascorbate pathway; (10) glyoxylate reductase. Reactions 1 and 2 can be catalyzed also by lactate dehydrogenase.

Isocitrate lyase is active in the glyoxylate cycle during germination of oil-storing seeds but its different form is present in green leaves where it interconverts glyoxylate, succinate and isocitrate (Igamberdiev et al., 1986; Eprintsev et al., 2015b). This form is magnesium-independent, operates at low pH (6-6.5), localized in cytosol, and can be activated by manganese. The role of isocitrate lyase in the accumulation of oxalate was shown already in 1960s in Atriplex (Osmond and Avadhani, 1968). It is possible that respiratory intermediates can serve as precursors of oxalate in plant leaves. Millerd et al. (1962, 1963) detected isocitrate lyase in the absence of malate synthase in Oxalis seedlings and indicated the possibility of its participation in the biogenesis of oxalate. Recently the isocitrate pathway was demonstrated as dominant to oxalate biosynthesis in sorrel (*Rumex obtusifolius*) (Miyagi et al., 2013). Glycolate oxidase (or one of its forms) still can play a role in oxalate biosynthesis by utilizing, in its side reaction, the glyoxylate formed by isocitrate lyase. Splitting of OAA to oxalate and acetate is another possible pathway of oxalate synthesis (Chang and Beevers, 1968; Franceschi and Nakata, 2005). One more source of oxalate in plant tissues can be via degradation of ascorbate (Franceschi and Nakata, 2005; Green and Fry, 2005), however, recent studies show minor if any involvement of this pathway (Yu et al., 2010). Pathways of oxalate metabolism are schematically shown on **Figure 6**.

**FIGURE 7:**
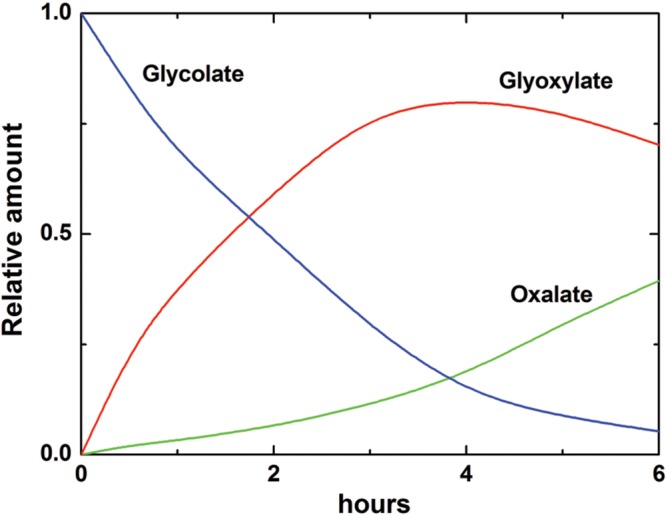
**Oxidation of glycolate and formation of glyoxylate and oxalate by glycolate oxidase from wheat leaves.** The changes of concentrations of glycolate, glyoxylate, and oxalate are calculated according to the kinetic equations based on the values of kinetic constants given in **Table 1**. Incorporation of the label from 1-^14^C-glycolate to glyoxylate and oxalate in the reaction catalyzed by the purified glycolate oxidase revealed a similar profile (Igamberdiev et al., 1988).

Accumulation of oxalate via glycolate oxidase can be explained by the kinetic properties of this enzyme, which exhibits the affinity not only to glycolate but also to its product glyoxylate, converting it to oxalate. Igamberdiev et al. (1988) studied the kinetics of glycolate oxidase isolated from wheat and sugar beet leaves and demonstrated that the affinity to glyoxylate of this enzyme is quite high (*K*_m_ ∼1 mM) (**Table 1**). **Figure 7** shows the rate of formation of glyoxylate and oxalate by the glycolate oxidase from wheat calculated from the obtained kinetic data and confirmed in the experiments on incorporation of the label from 1-^14^C-glycolate to glyoxylate and oxalate (Igamberdiev et al., 1988).

**Table 1:** Kinetic parameters of glycolate oxidase purified from wheat and sugar beet leaves (Igamberdiev et al., 1988).

Kinetic parameter	Wheat	Beet
*K*_m_ for glycolate, (mM)	0.22	0.37
*K*_m_ for glyoxylate (mM)	1.25	0.97
*V*_max_ for glycolate (μmol min^-1^ mg^-1^ protein)	9.2	6.2
*V*_max_ for glyoxylate (μmol min^-1^ mg^-1^ protein)	7.8	2.0
*K*_i_ for oxalate (mM), substrate glycolate	5.2	41
*K*_i_ for oxalate (mM), substrate glyoxylate	1.8	31

According to the Michaelis–Menten kinetics, the rate of oxidation of glycolate (and glyoxylate) is expressed by the following formula:

(3) $$dS/dt\;=\;VS/[S+K_{\text{m}}(1+S/K_{\text{m}})(1+I/K_{\text{i}})]$$

where *S* is the concentration of substrate (glycolate or glyoxylate), *V* is the maximal rate of oxidation of the corresponding substrate, *K*_m_ is the Michaelis constant for the corresponding substrate, *K*_i_ is the inhibitory constant for the corresponding substrate by oxalate, *I* – concentration of oxalate. The inhibition by glyoxylate is negligible and, therefore, not included in the equation. The value of *K*_i_ for oxalate for the wheat enzyme was much lower in both reactions than for the sugar beet enzyme. This means that oxalate inhibits its own accumulation in wheat leaves but not in sugar beet leaves. The data indicate that glycolate oxidase may represent an efficient tool for oxalate accumulation via the reaction of glyoxylate oxidation.

The role of glycolate and glyoxylate metabolism in oxalate accumulation was supported by the data of Fujii et al. (1993) and in more detail confirmed in the study of Yu et al. (2010). The authors observed that when glycolate or glyoxylate were fed into detached leaves, both organic acids effectively stimulated oxalate accumulation, which was completely inhibited by the glyoxylate scavenger cysteine. Oxalate accumulation, however, was not correlated with photorespiration. It is possible that glyoxylate as a precursor of oxalate originates in several reactions, including the cytosolic isocitrate lyase. The same authors (Yu et al., 2010) showed that the downregulation of L-galactono-1,4-lactone dehydrogenase, a key enzyme for ascorbate biosynthesis, did not affect oxalate levels, which makes ascorbate the unlikely precursor of oxalate. On the other hand, the peroxisomal glycolate oxidase may not be a major route of oxalate formation (Xu et al., 2006; Tian et al., 2008). There are several enzymes that can oxidize glycolate and glyoxylate in higher plants (Havir, 1983), however, further studies are needed to identify all these proteins. Some of them have been recently characterized, e.g., glycolate oxidase 3 in *Arabidopsis* which is homologous to yeast L-lactate: cytochrome *c* oxidoreductase and supports L-lactate oxidation in roots (Engqvist et al., 2015). Glycolate metabolizing enzymes share specificity and sequence similarity with lactate metabolizing enzymes (Engqvist et al., 2009, 2015). Glycolate, similarly to lactate (both 2-hydroxyacids), is a major organic acid accumulating under hypoxic conditions in rice (Narsai et al., 2009), and its formation can take place via the reduction of glyoxylate at high reduction levels observed in anaerobic environments.

Oxalate can be efficiently incorporated in metabolism (Zemlyanukhin et al., 1972; Havir, 1984; Ivanov et al., 1989); therefore it is not an end product. Its incorporation may take place through the transformation to oxalyl-CoA by oxalyl-CoA synthetase (Giovanelli, 1966). Both oxalyl-CoA synthetase and oxalate oxidase are usually connected with oxalate degradation; however, oxalyl-CoA can also be incorporated in biosynthetic reactions via glyoxylate (Schneider et al., 2012). The formation of glyoxylate from the labeled oxalate was demonstrated by Havir (1984). Ivanov et al. (1989) showed that, in pea leaves, the label from oxalate incorporated into glycine and serine, and at low levels into glycolate, alanine, glutamate, citrate, and malate.

Recent identification of oxalyl-CoA synthetase in *Arabidopsis* provides a support for the pathway of oxalate degradation first proposed by Giovanelli and Tobin (1961), but it also indicates a possibility of oxalate incorporation in metabolism. Foster et al. (2012, 2016) have established the role of this enzyme in oxalate catabolism in *Arabidopsis*, in the regulation of calcium oxalate crystal accumulation, and in the defense against oxalate-secreting phytopathogens in *Medicago truncatula*. In some plant species, but not *Arabidopsis*, oxalate is degraded by oxalate oxidase, which activity is exhibited by some germin proteins (Lane et al., 1993; Druka et al., 2002). This activity produces hydrogen peroxide in vacuoles and cell walls playing a role in hypersensitive responses to pathogen attack. Oxalate strongly inhibits NADPH-dependent cytosolic glyoxylate/ hydroxypyruvate reductase (Kleczkowski et al., 1991; Igamberdiev and Kleczkowski, 2000) thus suppressing the oxidation of cytosolic NADPH and scavenging of glyoxylate and hydroxypuruvate leaked to the cytosol.

**FIGURE 8:**
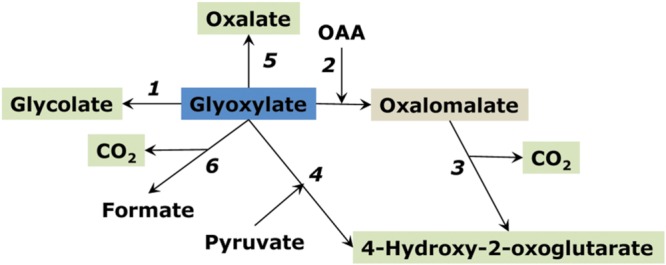
**Pathways of glyoxylate conversion in plants.** (1) reduction to glycolate; (2) non-enzymatic condensation with oxaloacetate (OAA) forming oxalomalate; (3) the latter is decarboxylated to 4-hydroxy-2-oxoglutarate; (4) enzymatic condensation with pyruvate forming 4-hydroxy-2-oxoglutarate; (5) oxidation to oxalate; (6) decarboxylation to formate.

Another organic acid, linked to the glycolate pathway and glyoxylate conversion, is formate. Its metabolism was reviewed in detail in Igamberdiev et al. (1999). Formate can be produced in the reaction of glyoxylate with hydrogen peroxide (Grodzinski and Butt, 1976; Wingler et al., 1999). It can be oxidized to CO_2_; however, formate dehydrogenase is induced mostly in heterotrophic tissues under stress conditions (Hourton-Cabassa et al., 1998). It is regulated via phosphorylation in a similar way as pyruvate dehydrogenase (Bykova et al., 2003) pointing possible glycolytic origin of formate in stressed heterotrophic tissues. However, the enzyme that converts pyruvate to formate (pyruvate: formate lyase) has not been yet identified in plants, it was identified only in Chlamydomonas among eukaryotic photosynthetic organisms (Atteia et al., 2006). Formate potentially could be formed via the reduction of CO_2_, the reaction which was considered for CO_2_ fixation before the discovery of the Calvin-Benson cycle (Baeyer, 1870). A small amount of formate possibly could be produced in photosynthetic electron transport chain (Kent, 1972); however, this reaction can have only very minor, if any, contribution to CO_2_ fixation. Condensation of two formate molecules can potentially yield glyoxylate which is further converted to glycine and serine, and then to other metabolites. This C_1_ pathway of photosynthesis was claimed for greening potato tubers (Ramaswamy and Nair, 1984) but never confirmed (for details see Igamberdiev et al., 1999). The link of glyoxylate to formate and other organic acids is shown on **Figure 8**.

## Hydroxyketoglutarate and Oxalomalate

Hydroxyketoglutarate (4-hydroxy-2-oxoglutarate) can be formed from glyoxylate and pyruvate by the enzyme 4-hydroxy-2-oxoglutarate aldolase (EC 4.1.3.16) (Kobes and Dekker, 1966). Oxalomalate is formed from glyoxylate and OAA by the enzyme oxalomalate lyase (EC 4.1.3.13); it may also be a side activity of 4-hydroxy-2-oxoglutarate aldolase (Scholtz and Schuster, 1984) (**Figure 8**). Decarboxylation of oxalomalate results in hydroxyketoglutarate formation (Ruffo et al., 1967; Adinolfi et al., 1969). Glyoxylate for hydroxyketoglutarate formation can originate from isocitrate lyase reaction (Morton and Wells, 1964).

Both oxalomalate and hydroxyketoglutarate are strong inhibitors of aconitase and isocitrate dehydrogenase (oxalomalate is one order of magnitude stronger inhibitor). Although the data on enzymatic formation and conversion of these acids in plants is very limited, it is known that hydroxyketoglutarate accumulates in several plants at significant concentrations, especially in oxalate accumulating plants, which provides an indirect evidence of glyoxylate as a precursor of both oxalate and hydroxyketoglutarate (Morton and Wells, 1964). The efficiency of oxalomalate and hydroxyketoglutarate and of glyoxylate itself in the inhibition of key enzymes of the TCA cycle may be important in the suppression of mitochondrial respiration during photorespiration, resulting in the citrate eﬄux from mitochondria and activation of the alternative oxidase (Gupta et al., 2012). Hydroxyketoglutarate aldolase reaction can be detected in several plants but with low activity (Igamberdiev, unpublished) and may appear as a result of the side activity of some aldolase with a broad specificity. Hydroxyketoglutarate can undergo oxidative decarboxylation with the formation of malate and reduction of NAD^+^ (Payes and Laties, 1963). The side reaction of malate dehydrogenase can result in the formation of another accumulating organic acid, L-2-hydroxyglutarate, which is oxidized to 2-oxoglutarate by L-2-hydroxyglutarate dehydrogenase (Hüdig et al., 2015).

## Excretion of Citrate, Malate and Oxalate and Weathering of Rocks

Organic acids in plants being the intermediates of photosynthetically driven carbon metabolism acquire several important physiological functions, one of which is related to their effects on the external environment. While vacuole represents the internalized external space being topologically equivalent to the cell exterior (Vitale and Denecke, 1999; Igamberdiev and Lea, 2002), the excretion of organic acids to the external medium is alternative to their vacuolar accumulation. In germinating cereal seeds, organic acids are excreted from scutellum and aleurone layer to endosperm to provide acidic conditions for breakdown of starch (Shchiparev et al., 1976; Ma et al., 2016). Roots can excrete diverse compounds of both primary and secondary metabolism, such as carbohydrates, carboxylates, phenolics and terpenoids, and the role of organic acids is especially important in building up of modern biosphere. Citrate and malate are the major excreted organic acids, and some plants excrete large quantities of oxalate (Meyer et al., 2010a).

Malate and citrate can be exported by three classes of transporters. Malate excretion at the plasma membrane occurs by aluminum-activated malate transporter (ALMT) channels, while it can also be excreted to the vacuole by tDT transporter. Citrate excretion is catalyzed by members of the MATE transporters. The detailed characterization of these transporters is given in the review of Meyer et al. (2010a). The excreted citrate, malate and oxalate can form complexes with micronutrients and toxic metals. At neutral pH, the stability constants between citrate and metals (Fe^3+^, Al^3+^, Zn^2+^ and Cd^2+^) are higher for citrate than for malate and oxalate, while at acidic pH, oxalate forms stronger complexes with metals than do malate and citrate due to its lower p*K* (Jones, 1998). The excretion of malate and citrate is very important for aluminum tolerance and for release of rock phosphate in cluster root forming plants, e.g., in white lupine (Massonneau et al., 2001). The aluminum tolerant cultivars of wheat have a greater ability to excrete malate from roots (Delhaize et al., 1993; de Andrade et al., 2011). In soybean cultivars, aluminum tolerance is associated with the excretion of citrate which sustained for longer period than the excretion of malate (Silva et al., 2001). Citrate plays also an important role for iron acquisition and transport within the plant (Durrett et al., 2007).

Excretion of citrate and malate is related to the intensity of enzymes of the two branches of the TCA cycle, while the mechanisms of oxalate excretion are less certain. The detailed studies have been performed to elucidate the mechanism of excretion of citrate. Overexpression of *Arabidopsis* citrate synthase in carrot cells improved the growth in aluminum phosphate medium as a result of citrate excretion, which was three to four times greater as compared to control (Koyama et al., 1999). When the gene for mitochondrial citrate synthase from carrot was introduced into *Arabidopsis*, a 2.5-fold increase in excretion of citrate was observed with a correlation between the levels of citrate synthase activity and the amounts of citrate excreted into the medium (Koyama et al., 2000). The overexpression of citrate synthase improved the growth in phosphorus limited soil as a result of enhanced citrate excretion from the roots. Aluminum tolerance also increased in transgenic canola overexpressing citrate synthase (Anoop et al., 2003). Overexpression of the mitochondrial malate dehydrogenase improved phosphorus acquisition by tobacco plants (Lü et al., 2012).

The role of excretion of organic acids is globally biospheric. It is considered as the main cause of soil formation via weathering of rocks starting from the Ordovician period, when bryophytes colonized land (Lenton et al., 2012), and then continuing with the rooted plants in Silurian and Devonian (Raven and Edwards, 2001; Igamberdiev and Lea, 2006). The decrease in the atmospheric CO_2_ during these times is also attributed to weathering of rocks when calcium silicates were replaced by carbonates (Berner, 1993, 1997). Although the regulation of atmospheric CO_2_ via the influence on weathering of rocks is attributed to the heterotrophic metabolism of roots, it uses photosynthetically fixed carbon and therefore complements the direct regulation of CO_2_/O_2_ ratio by photosynthesis and photorespiration (Tolbert et al., 1995).

## Conclusion

Organic acids accumulate in plants mainly as a result of the incomplete oxidation of photosynthetic products and represent the stored pools of fixed carbon due to different transient times of conversion of carbon compounds in metabolic pathways. The increase in the redox level in the cell in the conditions of active photosynthesis results in the transformation of the TCA cycle to an open branched structure supplying citrate for the synthesis of 2-oxoglutarate and glutamate (citrate valve), while accumulation of malate regulates the redox balance in different cell compartments (via malate valve). Besides the organic acids – intermediates of the TCA cycle, several carboxylates are linked with photorespiration, catabolism of amino acids and other processes. The intermediates of the TCA cycle can be transformed to the secondary organic acids that form the additional pools of fixed carbon and stabilize the operation of TCA cycle and regulate redox balance. Metabolism of the secondary organic acid such as hydroxycitrate, *trans*-aconitate, hydroxyketoglutarate can be related to the side activities of known enzymes, however, in most cases it involves several plant enzymes that are not well characterized, such as citrate dehydrase, aconitate isomerase, putative hydroxycitrate dehydratase (decarboxylating), or hydroxyketoglutarate aldolase. Although the activities of these enzymes were detected and some of them are partially purified and characterized, they are not studied at the genetic level, and the knowledge about them is very incomplete. Further studies in this direction are important to clarify the specificity of plant carbon metabolism and to identify novel metabolic pathways operating in photosynthetic organisms.

## Author Contributions

All authors listed, have made substantial, direct and intellectual contribution to the work, and approved it for publication.

## Conflict of Interest Statement

The authors declare that the research was conducted in the absence of any commercial or financial relationships that could be construed as a potential conflict of interest.
